# Burnout and its association with extracurricular activities among medical students in Saudi Arabia

**DOI:** 10.5116/ijme.58e3.ca8a

**Published:** 2017-04-26

**Authors:** Sami A. Almalki, Abdullah I. Almojali, Ali S. Alothman, Emad M. Masuadi, Meshal K. Alaqeel

**Affiliations:** 1 College of Medicine, King Saud bin Abdulaziz University for Health Sciences, Riyadh, Saudi Arabia; 2 Department of Medical Education, King Saud bin Abdulaziz University for Health Sciences, Riyadh, Saudi Arabia; 3 Psychiatry Department, King Abdulaziz Medical City, Riyadh, Saudi Arabia

**Keywords:** Burnout, extracurricular activities, medical education, medical students, Saudi Arabia

## Abstract

**Objectives:**

To assess levels of burnout in medical students, and to explore the influence of extracurricular activities on burnout at a medical school in Saudi Arabia.

**Methods:**

This cross-sectional study was conducted with first to fourth year medical students at King Saud bin Abdulaziz University for Health Sciences (KSAU-HS) in Riyadh, Saudi Arabia. Socio-demographic, burnout level (the Maslach Burnout Inventory-Student Survey, MBI-SS) and participation in extracurricular activities data were collected using a self-administered questionnaire. Statistical analyses were performed using the Pearson’s chi-square test and binary logistic regression.

**Results:**

From the 306 medical students approached, 249 (81.4%) completed the questionnaire. The level of high burnout was 67.1% (n=167). The study revealed that the majority (62.3%, n=155) of students had high levels of cynicism, 58.6% (n=146) had high levels of emotional exhaustion, and 60.2% (n=150) had low levels of professional efficacy. Most of the students (73.5%, n=183) participated in extracurricular activities, and 112 (45%) students were organizers of extracurricular activities. No significant association was found between burnout levels and the frequency of involvement in extracurricular activities (χ^2^=2.2, df=2, p=0.333). However, students who were organizing extracurricular activities were less likely to have low professional efficacy (OR=0.51, 95% CI: 0.27- 0.96).

**Conclusions:**

High levels of burnout were reported at this medical school. Although the burnout level is not significantly associated with the frequency of involvement in extracurricular activities, leading and organizing extracurricular activities might result in lower levels of burnout. Therefore, improving the students’ leadership skills should be considered in curriculum planning, and greater emphasis should be placed on the quality of involvement in extracurricular activities rather than the quantity.

## Introduction

Medical schools are devoted to establishing an enabling learning environment to develop highly qualified physicians having the required knowledge, skills and competence. Medical students go through stressful events, and they are engaged in multiple activities. They experience academic, existential and psychological stressors that may cause deteriorating mental health during medical school.^1^ Studies have shown that health related quality of life is poor among medical students,^2^ and students in their clinical years experience the most negative impact.^3^ Muneer and colleagues^4^ reported high levels of stress among Malaysian dental students, with fear of failure in a course being ranked as the top stressor. Consequently, students who experience a high level of stress with poor coping strategies during medical school are vulnerable to develop burnout.^5^^-^^7^

Burnout is a mental condition defined as a prolonged response to chronic emotional and interpersonal stressors on the job.^8^ It is characterized by a triad of emotional exhaustion, cynicism and a feeling of personal inefficacy. Studies have shown that the burnout level is high among medical students, ranging from 45% to 50%.^1^^,^^9^^-^^11^ In studies done in Lebanon and the United States, burnout was found to be higher among pre-clinical students (71%).^12^^,^^13^ Locally in Saudi Arabia, only one study could be found reporting a very high burnout level (76.8%).^14^

Burnout is an independent risk factor for students’ suicidal ideation and can lead to dropping out of medical school.^9^ This is due to students performing many different tasks as well as starting to clinically care for patients with a lack of knowledge and experience, resulting in an increased level of stress. Students’ mental health should receive more attention from medical educators and health care personnel. Hojat and colleagues^15^ found higher optimism levels to be associated with higher personal accomplishment and lower emotional exhaustion among third-year medical students. Physical activity also has been reported as the best lifestyle factor to reduce burnout, and a program of increased healthy physical activities can lead to an improvement in burnout.^16^^,^^17^ Striking a balance between academic commitments and relaxation may positively influence quality of life and prevent burnout.^18^^,^^19^

Extracurricular activities provide students the opportunity to experience nonacademic activities that could be a powerful strategy for managing stress and bringing their life into balance.^12^ Motivation to participate in extracurricular activities are divided into two coexisting classes.^20^ The first is altruistic or values based in nature which includes helping others, religious beliefs and supporting an important value. The second category involves utilitarian motives such as developing a new skill set and enhancing resumes. In a cross-national study aimed to examine motivations to volunteer of more than 9000 college students from diverse disciplines from 12 countries, altruistic and value based motives were the strongest support for volunteering.^21^ The previous study showed a lower intensity and regularity of volunteering in students who were more strongly motivated by resume building.

Benefits of involvement in extracurricular activities are well documented. For example, leadership ability, critical thinking, social self-confidence and conflict resolution skills were found higher in students who volunteer.^22^ Undergraduate voluntary participation can positively affect educational outcomes such as the acquisition of higher degrees.^23^ In fact, it was reported that students who reduce their recreation had, on average, lower examination results.^24^ Lumley et al. reported a diversity of extracurricular activities among final year medical students.^25^ They found that students, on average, spent 9.8 hours per week in extracurricular activities. Students who engaged in research and teaching as extracurricular activities achieved higher academic performance. Additionally, high educational aspiration is associated with consistent involvement in extracurricular activities, which may afford opportunities to build interpersonal skills and construct positive plans for the future.^23^ In a large longitudinal study, the total number of extracurricular activities was positively associated with psychological resilience, school belonging, and academic peers compared to being negatively related to risky peers and psychological distress.^26^

In view of the literature, it is crucial to have a better understanding of students’ mental health as it has implications for all involved in their welfare and education. Therefore, this study aimed to assess the levels of burnout and to explore the influence of extracurricular activities on burnout in medical students at a medical school in Saudi Arabia.

## Methods

### Study design and participants

This cross-sectional study was conducted with first to fourth year medical students from King Saud bin Abdulaziz University for Health Sciences (KSAU-HS) in Riyadh, Saudi Arabia. Of 756 medical students, 306 students were invited to participate in the study. The questionnaire was completed by 249 students, resulting in an 81.4% response rate. A third (32.5%, n=81) of the participants were female. Ages ranged between 19-30 years with a mean age of 21.8 (SD=1.4). Participation was entirely voluntary and responses were anonymized. This study was approved by the Institutional Review Board of King Abdullah International Medical Research Center.

### Instrument

The questionnaire consisted of three sections. Section I identified socio-demographics and personal characteristics of the participants such as age, gender, academic year, current grade point average (GPA), having a physician among first-degree family members, smoking and marital status. In KSAU-HS, the cumulative grade point average (GPA) is out of five and can be described as a grade (e.g. GPAs 4.50 and above are considered as excellent).

In Section II, the Maslach Burnout Inventory-Student Survey (MBI-SS) – the modified version of Maslach Burnout Inventory-General Survey (MBI-GS) – was used to assess burnout among the participants. It has been validated by Schaufeli demonstrating adequate reliability among Spanish, Portuguese, and Dutch students.^27^ The MBI-SS has been shown to have adequate reliability and factorial validity among Chinese students.^28^ It consists of 16 questions focusing on the triad of exhaustion (5 items), cynicism (5 items), and professional efficacy (6 items). Exhaustion refers to severe fatigue and emotional depletion due to academic demands; cynicism refers to the student’s mental distance from lessons; and professional efficacy refers to academic accomplishment. All items were assessed by a seven-point Likert scale ranging from 0 (Never) to 6 (Always). Scores in each of the three components were categorized into high, moderate or low scores. High scores on cynicism, exhaustion, and low scores on professional efficacy (professional efficacy items are reverse scored) indicate high burnout.^28^ In this study, the Cronbach’s alpha value for the MBI-SS was 0.75 for professional efficacy, 0.85 for exhaustion and 0.76 for cynicism.

In Section III, questions targeted the frequency and the type of involvement in extracurricular activities during the past three months. Extracurricular activities are voluntary activities external to the curriculum. In our university, many of the extra academic activities such as volunteer/community services, research and reading activities, and arts and cultural activities are performed by students through the Students Club.

**Table 1:** Socio-demographics and personal characteristics of the study participants (n=249)

Variable		n	%
Gender			
	Male	168	67.5
	Female	81	32.5
Age (years)			
	20-23	200	81.3
	≥24	46	18.7
Grade point average (out of 5)			
	<4.50	103	42.4
	4.50–5.00	140	57.6
Academic year			
	1^st^ year	97	39.0
	2^nd^ year	58	23.3
	3^rd^ year	50	20.1
	4^th^ year	44	17.6
Stream			
	Stream I (undergraduates)	222	89.2
	Stream II (postgraduates)	27	10.8
Physician in family			
	No	133	53.4
	Yes	116	46.6
The frequency of involvement in extracurricular activities^†^			
	None	66	26.5
	1-3	105	42.2
	≥ 4	78	31.3
Organizing extracurricular activities (during the past three months)			
	No	137	55.0
	Yes	112	45.0
Type of reported extracurricular activities			
	Volunteer/Community services	122	49.2
	Research and reading activities	110	44.4
	Arts and cultural activities	88	35.5

### Procedures

The study was conducted in March and April 2016 using a self-administered questionnaire. The study’s objectives, primary investigator’s contact details and the ethical principles guiding the study were provided and informed written consent was obtained. A week was assigned to collect data from each academic year and data were collected at least two weeks prior to any examination date in order to minimize short-term external stressors that may erroneously affect the burnout level. The students completed the questionnaire during their breaks between classes and returned it in a sealed envelope. All gathered information was kept confidential.

### Statistical analysis

Data were entered in Microsoft Excel and analyzed by IBM-SPSS software (version 22.0). Categorical variables were presented by frequencies and percentages. The association of burnout with extracurricular activities and demographics was examined using the Pearson’s chi-square test. Binary logistic regression was done to identify significant determinants of burnout components controlling for potential cofounders. The burnout subscales (professional efficacy, exhaustion and cynicism) were used as dependent variables, while other socio-demographic variables and extracurricular activities were entered as predictor variables. Odds ratios (OR) and 95% confidence intervals (95% CI) were calculated. A test with a p-value <0.05 was considered as statistically significant.

**Table 2:** Levels of burnout and its components among medical students of King Saud bin Abdulaziz University for Health Sciences (n=249)

Level	Professional efficacy		Exhaustion		Cynicism		Burnout	
	n	%	n	%	n	%	n	%
High	32	12.9	146	58.6	155	62.3	167	67.1
Moderate	67	26.9	56	22.5	67	26.9	51	20.5
Low	150	60.2	47	18.9	27	10.8	31	12.4

## Results

As shown in Table 1, most of the students (73.5%, n=183) were involved in extracurricular activities during the past three months. Volunteer/community services were the most frequently reported extracurricular activity (49.2%, n=122) followed by research and reading activities (44.4%, n=110), and arts and cultural activities (35.5%, n=88). Almost half of the students (45%, n=112) were leading and organizing extracurricular activities in the past three months. Most of the students (96.4%, n=240) were single, 230 (92.4%) lived with their families and 228 (91.9%) were non-smokers. Almost half of the students (46.6%, n=116) had a physician among their family members. There were 160 (64.5%) students who had conducted some research, while only 23 (9.3%) of the participants published their research. Table 2 shows that 62.3% (n=155) of the students had high levels of cynicism, 58.6% (n=146) had high levels of emotional exhaustion, and 60.2% (n=150) had low levels of professional efficacy.

The association between burnout and its components with socio-demographic characteristics and the involvement in extracurricular activities is shown in Table 3. Students who achieve a grade point average (GPA) <4.5 showed significantly low professional efficacy (χ^2^=7.189, df=1, p=0.007). Female students were found to have significantly low levels of cynicism (χ^2^=10.113, df=2, p=0.006, n=16) and low burnout (χ^2^=6.045, df=2, p=0.049, n=16) compared to males. Third year medical students showed lower levels of cynicism compared to the other years (χ^2^=9.575, df=3, p=0.023). Students who have a physician among family members showed significantly high exhaustion (χ^2^=5.371, df=1, p=0.002) and high cynicism (χ^2^=7.998, df=1, p=0.005). Students who did not lead or organize extracurricular activities showed significantly low professional efficacy (χ^2^=7.427, df=1, p=0.006). There was no significant association between burnout and the frequency of involvement in extracurricular activities (χ^2^=2.2, df=2, p=0.333). Undergraduates and postgraduates did not show a significant difference in burnout levels (χ^2^=0.231, df=1, p=0.631).

**Table 3:** Association between burnout and burnout components with socio-demographic characteristics and the involvement in extracurricular activities (n=249)

Variable		Count	Low PE		High EX		High CY		High Burnout	
			n	%	n	%	n	%	n	%
Gender	Male	168	105	62.5	96	57.1	108	64.3	116	69.0
	Female	81	45	55.6	50	61.7	47	58.0	51	63.0
	p-value		0.29		0.49		0.34		0.34	
Age (years)	20-23	200	120	60.0	116	58.0	121	60.5	131	65.5
	≥24	46	28	60.9	27	58.7	31	67.4	33	71.7
	p-value		0.91		0.93		0.39		0.42	
Grade point average (out of 5)	<4.50	103	72	69.9	62	60.2	65	63.1	73	70.9
	≥4.50	140	74	52.9	79	56.4	85	60.7	88	62.9
	p-value		0.007^*^		0.56		0.70		0.19	
Academic year	1^st^ year	97	54	55.7	64	66.0	62	63.9	64	66.0
	2^nd^ year	58	35	60.3	32	55.2	40	69.0	40	69.0
	3^rd^ year	50	34	68.0	25	50.0	22	44.0	31	62.0
	4^th^ year	44	27	61.4	25	56.8	31	70.5	32	72.7
	p-value		0.55		0.26		0.02^*^		0.71	
Stream	I	222	135	60.8	132	59.5	138	62.2	150	67.6
	II	27	15	55.6	14	51.9	17	63.0	17	63.0
	p-value		0.60		0.45		0.93		0.63	
Physician in family	No	133	82	61.7	69	51.9	72	54.1	87	65.4
	Yes	116	68	58.6	77	66.4	83	71.6	80	69.0
	p-value		0.63		0.02^*^		0.005^*^		0.55	
The frequency of involvement in extracurricular activities^†^	None	66	47	71.2	41	62.1	44	66.7	47	71.2
	1-3	105	62	59.0	57	54.3	64	61.0	65	61.9
	≥4	78	41	52.6	48	61.5	47	60.3	55	70.5
	p-value		0.07		0.49		0.69		0.33	
Organizing extracurricular activities^†^	No	137	93	67.9	82	59.9	86	62.8	92	67.2
	Yes	112	57	50.9	64	57.1	69	61.6	75	67.0
	p-value		0.006^*^		0.67		0.85		0.97	

Key: PE - professional efficacy; EX – exhaustion; CY - cynicism Stream I – undergraduates; Stream II – postgraduates †During the past three months *Significant at p value < 0.05; p values were calculated using the Pearson chi-square

Students who have a high GPA (≥ 4.5) were more involved in participating (χ^2^=19.213, df=2, p<0.001) and organizing (χ^2^=5.259, df=1, p=0.022) extracurricular activities compared to those with a GPA of < 4.5. Volunteering in community services (χ^2^=9.125, df=1, p=0.003) and participating in arts and cultural activities (χ^2^=6.864, df=1, p=0.009) were more frequent among females compared to males. 

Binary logistic regression was conducted to determine the association between the burnout components as dependent variables and demographics and the extracurricular activities as independent variables. Table 4 shows that having a GPA that is <4.5 was significantly associated with low professional efficacy (OR=1.99, 95% CI: 1.10-3.60). Having a physician among family members was a significant predictor of high emotional exhaustion (OR=1.86, 95% CI: 1.08-3.19) and high cynicism (OR=2.30, 95% CI: 1.31-4.05). Students who were leading and organizing extracurricular activities were less likely to have low professional efficacy (OR=0.51, 95% CI: 0.27-0.96).

## Discussion

The current study is one of a few studies that explores the magnitude of burnout among medical students in Saudi Arabia, and it is the first one to investigate the role of extracurricular activities in burnout. The results of this study show alarming findings and reveal a high burnout level (67%) among medical students at a university in Saudi Arabia. Literature reports a wide range of burnout levels among medical students from different countries. Our results are comparable with recent studies related to burnout ranging between 71% and 76.8%.^12^^-^^14^ In contrast, numerous studies reported lower burnout levels ranging between 10.3% and 55%.^1^^,^^10^^,^^11^^,^^29^^-^^31^ These varying levels could be explained by the different instruments used to assess burnout since there is no standard instrument or due to different underlying causes of burnout.

However, the high level of burnout among medical students in Saudi Arabia is a cause of concern as it can adversely affect the learning process and ultimately reduce the quality of health care delivered to patients.^5^ The high levels found in our study could be attributed to a variety of factors. One of which is the high level of competition among students in Saudi Arabia to enrol in medical school, or to be accepted in a desired residency program due to the high number of graduates each year and the lack of residency positions. Students have to build impressive resumes with published research, presenting in conferences, health promotion activities and many other requirements play a significant role in being accepted. 

The results show that the frequency of attendance in extracurricular activities does not have a significant influence on burnout. This contradicts a previous study with high school students where students who participated in a greater number of extracurricular activities reported a subjective feeling of well-being.^32^ Additionally, volunteers who are a part of the workforce showed lower levels of burnout, stress and higher levels of psychological well-being.^33^ Our results may imply that the objective of participating in extracurricular activities is not related to students’ well-being as a priority. While helping the community as a part of extracurricular activities is essential, medical educators and university officials should consider engaging students more in activities with the aim of alleviating stressors among students.

**Table 4:** Outcome of the logistic regression analysis for burnout components and the study variables (n=249)

Variable		Low professional efficacy			High exhaustion			High cynicism		
		OR	95%CI	p-value	OR	95%CI	p-value	OR	95%CI	p-value
Gender										
	Male	1.25	(0.70, 2.23)	0.45	0.85	(0.48,1.51)	0.58	1.46	(0.82, 2.62)	0.20
	Female^†^	1			1			1		
Age (years)										
	20-23	0.85	(0.24, 2.97)	0.79	0.41	(0.12,1.40)	0.16	0.59	(0.17, 2.11)	0.42
	≥ 24^†^	1			1			1		
Grade point average										
	< 4.50	1.99	(1.10, 3.60)	0.02^*^	1.21	(0.68, 2.13)	0.52	1.01	(0.56,1.82)	0.97
	≥4.50^†^	1			1			1		
Academic year										
	1^st^ year	0.99	(0.39, 2.49)	0.98	2.26	(0.92, 5.57)	0.08	0.98	(0.39, 2.46)	0.96
	2^nd^ year	1.42	(0.52, 3.84)	0.49	1.47	(0.56, 3.86)	0.43	1.45	(0.53, 3.98)	0.47
	3^rd^ year	1.66	(0.59, 4.64)	0.33	1.23	(0.47, 3.26)	0.67	0.44	(0.17,1.19)	0.11
	4^th^ year^†^	1			1			1		
Stream										
	Stream I	2.46	(0.57, 10.55)	0.23	3.02	(0.72,12.69)	0.13	1.37	(0.31, 6.09)	0.68
	Stream II^†^	1			1			1		
Physician in family										
	Yes	0.74	(0.42, 1.28)	0.28	1.86	(1.08, 3.19)	0.03^*^	2.30	(1.31, 4.05)	0.004^*^
	No^†^	1			1			1		
The frequency of involvement in extracurricular activities^‡^										
	1-3	1.54	(0.64, 3.73)	0.34	0.82	(0.35, 1.94)	0.65	1.31	(0.54, 3.16)	0.55
	≥ 4	1.14	(0.58, 2.27)	0.70	0.69	(0.35, 1.36)	0.28	1.09	(0.55, 2.18)	0.81
	None^†^	1			1			1		
Organizing extracurricular activities^‡^										
	Yes	0.51	(0.27, 0.96)	0.04^*^	0.91	(0.49, 1.70)	0.76	1.12	(0.59, 2.12)	0.74
	No^†^	1			1			1		

Key: Stream I – undergraduates; Stream II – postgraduates; OR - Odds ratio, CI - Confidence interval *Significant at p value < 0.05 †Reference group; ‡During the past three months

Our results showed that students who were involved as leaders or organizers of the extracurricular activities reported a better professional efficacy. This may be interpreted that deep involvement is more influential as students   have an opportunity to improve their leadership abilities, which may positively reflect in their academic performance. The assumption is supported by a previous study indicating that previous student leadership experience could positively affect future organizational involvement.^34^ Being an organizer can enable the student to form a strong connection with academically oriented peers who share similar interests, and as a result, they can assist each other.

The current study showed females to have lower levels of burnout, which is supported by the findings in two previous studies where male students were at higher risk of burnout than their female counterparts.^16^^,^^29^ However, other studies published contradictory results as they reported that females suffered burnout more than males.^10^^,^^12^ The lower female burnout result in the current study could be attributed to higher financial and social pressures encountered by males in Saudi Arabia.  Furthermore, female participation in extracurricular activities was significantly higher than the males. This finding is consistent with another study that showed greater participation in extracurricular activities by females and more time spent in medical volunteering.^35^ However, the design of this study limits detailed analysis of gender differences. Further studies are needed to investigate these differences.

An interesting finding of the present study is that students who have a physician among family members show significantly high emotional exhaustion and high cynicism. The finding is supported in literature with a study reporting that students whose parents are physicians showed high burnout.^10^ The finding appears paradoxical as the assumption is that these students could be guided by their parents or siblings and therefore have a more positive psychological status. In fact, parents who are physicians have been reported as the second highest source of stress in a previous medical student related study.^36^ This can be due to the high expectations by parents or siblings, which could result in experiencing more pressure, thus causing a negative psychological status among students.

Our findings demonstrate that students who achieve a low GPA (<4.50) have low professional efficacy resulting in higher burnout levels. The low GPA serves as an additional source of stress as students need to improve their GPA since it plays such a major role in their future careers. A recommendation from the current study is that medical educators should not only provide support and guidance but also focus on this issue in terms of policy reform and curricular changes. Possible examples include changing the method of grading from a letter-grade hierarchical system to pass/fail system as numerous studies showed that such a change resulted in lower burnout levels, lower stress and higher psychological well-being without affecting academic performance.^37^^-^^39^

### Limitations of the study

There are several limitations inherent in this study. First, this study is limited by its cross-sectional design which cannot determine causal relationships. Additionally, some degree of recall or response bias such as social desirability^40^ can arise due to using a self-administered questionnaire. Finally, the current study was conducted in one college, and while these results might be generalizable to other local medical colleges, future research might investigate this across different medical schools.

## Conclusions

The students reported higher levels of burnout. Although there was no significant association between the frequency of participation in extracurricular activities and burnout, this study showed that leading and organizing extracurricular activities might positively affect students’ level burnout. Based on these findings, students’ mental health should receive more attention from medical educators and health care personnel. Preventive measures such as improving coping skills, focused support, and increased mental health awareness should be considered. Additionally, improving the leadership skills of students should be embedded in curriculum planning and assessment, and greater emphasis should be placed on the quality of involvement in extracurricular activities rather than the quantity. Further longitudinal studies are required to study the pattern of burnout among medical students from admission to medical schools until graduation.

### Acknowledgments

The authors gratefully acknowledge Bashaier Alenizy, Norah Alsabty, Futun Aljoufi and Alanoud Aldrees who assisted in the data collection for this project. Special thanks to Dr. Sultan Almalki, Dr. Aamir Omair and Mr. Sulaiman Jenkins for revising and editing the manuscript.

### Conflict of Interest

The authors declare that they have no conflict of interest.

## References

[1] Dyrbye LN, Thomas MR, Huntington JL, Lawson KL, Novotny PJ, Sloan JA, Shanafelt TD (2006). Personal life events and medical student burnout: a multicenter study.. Acad Med.

[2] Lins L, Carvalho FM, Menezes MS, Porto-Silva L, Damasceno H (2015). Health-related quality of life of students from a private medical school in Brazil.. Int J Med Educ.

[3] Paro HB, Morales NM, Silva CH, Rezende CH, Pinto RM, Morales RR, Mendonça TM, Prado MM (2010). Health-related quality of life of medical students.. Med Educ.

[4] Babar MG, Hasan SS, Ooi YJ, Ahmed SI, Wong PS, Ahmad SF, Mnm-Rosdy NM, Malik NA (2015). Perceived sources of stress among Malaysian dental students.. Int J Med Educ.

[5] Ishak W, Nikravesh R, Lederer S, Perry R, Ogunyemi D, Bernstein C (2013). Burnout in medical students: a systematic review.. Clin Teach.

[6] Humphris G, Blinkhorn A, Freeman R, Gorter R, Hoad-Reddick G, Murtomaa H, O'Sullivan R, Splieth C (2002). Psychological stress in undergraduate dental students: baseline results from seven European dental schools.. Eur J Dent Educ.

[7] Pöhlmann K, Jonas I, Ruf S, Harzer W (2005). Stress, burnout and health in the clinical period of dental education.. Eur J Dent Educ.

[8] Maslach C, Schaufeli WB, Leiter MP (2001). Job burnout.. Annu Rev Psychol.

[9] Dyrbye LN, Thomas MR, Massie FS, Power DV, Eacker A, Harper W, Durning S, Moutier C, Szydlo DW, Novotny PJ, Sloan JA, Shanafelt TD (2008). Burnout and suicidal ideation among U.S. medical students.. Ann Intern Med.

[10] Muzafar Y, Khan HH, Ashraf H, Hussain W, Sajid H, Tahir M, Rehman A, Sohail A, Waqas A, Ahmad W (2015). Burnout and its associated factors in medical students of Lahore, Pakistan.. Cureus.

[11] Dahlin ME, Runeson B (2007). Burnout and psychiatric morbidity among medical students entering clinical training: a three year prospective questionnaire and interview-based study.. BMC Med Educ.

[12] Fares J, Saadeddin Z, Al Tabosh H, Aridi H, El Mouhayyar C, Koleilat MK, Chaaya M, El Asmar K (2016). Extracurricular activities associated with stress and burnout in preclinical medical students.. J Epidemiol Glob Health.

[13] Mazurkiewicz R, Korenstein D, Fallar R, Ripp J (2012). The prevalence and correlations of medical student burnout in the pre-clinical years: a cross-sectional study.. Psychol Health Med.

[14] El-Masry R, Ghreiz SM, Helal RM, Audeh AM, Shams T. Perceived stress and burnout among medical students during the clinical period of their education. Ibnosina J Med Biomed Sci. 2013;5(4):179-87.

[15] Hojat M, Vergare M, Isenberg G, Cohen M, Spandorfer J (2015). Underlying construct of empathy, optimism, and burnout in medical students.. Int J Med Educ.

[16] Cecil J, McHale C, Hart J, Laidlaw A (2014). Behaviour and burnout in medical students.. Med Educ Online.

[17] Gerber M, Brand S, Elliot C, Holsboer-Trachsler E, Pühse U, Beck J (2013). Aerobic exercise training and burnout: a pilot study with male participants suffering from burnout.. BMC Res Notes.

[18] Zhang Y, Qu B, Lun S, Wang D, Guo Y, Liu J (2012). Quality of life of medical students in China: a study using the WHOQOL-BREF.. PLoS ONE.

[19] Henning MA, Hawken SJ, Hill AG (2009). The quality of life of New Zealand doctors and medical students: what can be done to avoid burnout?. N Z Med J.

[20] Cnaan RA, Goldberg-Glen RS. Measuring motivation to volunteer in human services. Journal of Applied Behavioral Science. 1991; 27(3):269-84.

[21] Handy F, Cnaan RA, Hustinx L, Kang C, Brudney JL, Haski-Leventhal D, Holmes K, Meijs LCPM, Pessi AB, Ranade B, Yamauchi N, Zrinscak S (2010). A cross-cultural examination of student volunteering: is it all about resume building?. Nonprofit and Voluntary Sector Quarterly.

[22] Astin A, Sax L, Avalos J. Long-term effects of volunteerism during the undergraduate years. The Review of Higher Education. 1999; 22(2):187-202.

[23] Mahoney JL, Cairns BD, Farmer TW. Promoting interpersonal competence and educational success through extracurricular activity participation. J Educ Psychol. 2003;95(2):409-18.

[24] Slade AN, Kies SM (2015). The relationship between academic performance and recreation use among first-year medical students.. Med Educ Online.

[25] Lumley S, Ward P, Roberts L, Mann JP (2015). Self-reported extracurricular activity, academic success, and quality of life in UK medical students.. Int J Med Educ.

[26] Fredricks JA, Eccles JS (2006). Extracurricular involvement and adolescent adjustment: impact of duration, number of activities, and breadth of participation.. Applied Developmental Science.

[27] Schaufeli WB, Martinez IM, Pinto AM, Salanova M, Bakker AB (2002). Burnout and engagement in university students: a cross-national study.. Journal of Cross-Cultural Psychology.

[28] Hu Q, Schaufeli WB (2009). The factorial validity of the Maslach Burnout Inventory-Student Survey in China.. Psychol Rep.

[29] Costa EF, Santos SA, Santos AT, Melo EV, Andrade TM (2012). Burnout Syndrome and associated factors among medical students: a cross-sectional study.. Clinics (Sao Paulo).

[30] Youssef FF (2016). Medical student stress, burnout and depression in Trinidad and Tobago.. Acad Psychiatry.

[31] Chang E, Eddins-Folensbee F, Coverdale J (2012). Survey of the prevalence of burnout, stress, depression, and the use of supports by medical students at one school.. Acad Psychiatry.

[32] Palen LA, Coatsworth JD (2007). Activity-based identity experiences and their relations to problem behavior and psychological well-being in adolescence.. J Adolesc.

[33] Ramos R, Brauchli R, Bauer G, Wehner T, Hämmig O (2015). Busy yet socially engaged: volunteering, work-life balance, and health in the working population.. J Occup Environ Med.

[34] Schuh JH, Laverty M. The perceived long-term influence of holding a significant student leadership position. J Coll Stud Pers. 1983; 24: 28-32.

[35] Kim SH (2016). Extracurricular activities of medical school applicants.. Korean J Med Educ.

[36] Sreeramareddy CT, Shankar PR, Binu VS, Mukhopadhyay C, Ray B, Menezes RG (2007). Psychological morbidity, sources of stress and coping strategies among undergraduate medical students of Nepal.. BMC Med Educ.

[37] Reed DA, Shanafelt TD, Satele DW, Power DV, Eacker A, Harper W, Moutier C, Durning S, Massie FS, Thomas MR, Sloan JA, Dyrbye LN (2011). Relationship of pass/fail grading and curriculum structure with well-being among preclinical medical students: a multi-institutional study.. Acad Med.

[38] Bloodgood RA, Short JG, Jackson JM, Martindale JR (2009). A change to pass/fail grading in the first two years at one medical school results in improved psychological well-being.. Acad Med.

[39] Rohe DE, Barrier PA, Clark MM, Cook DA, Vickers KS, Decker PA (2006). The benefits of pass-fail grading on stress, mood, and group cohesion in medical students.. Mayo Clin Proc.

[40] Tourangeau R, Yan T (2007). Sensitive questions in surveys.. Psychol Bull.

